# Regulation of Enteric Infection and Immunity by Dietary Proanthocyanidins

**DOI:** 10.3389/fimmu.2021.637603

**Published:** 2021-02-24

**Authors:** Audrey I. S. Andersen-Civil, Pankaj Arora, Andrew R. Williams

**Affiliations:** Department of Veterinary and Animal Sciences, University of Copenhagen, Faculty of Health and Medical Sciences, Frederiksberg, Denmark

**Keywords:** pathogens, proanthocyanidins, mucosal immunity, microbiota, enteric infection, inflammation

## Abstract

The role of dietary components in immune function has acquired considerable attention in recent years. An important focus area is to unravel the role of bioactive dietary compounds in relation to enteric disease and their impact on gut mucosal immunity. Proanthocyanidins (PAC) are among the most common and most consumed dietary polyphenols, and are characterised by their variable molecular structures and diverse bioactivities. In particular, their anti-oxidative effects and ability to modulate gut microbiota have been widely described. However, there is limited evidence on the mechanism of action of PAC on the immune system, nor is it clearly established how PAC may influence susceptibility to enteric infections. Establishing the sites of action of PAC and their metabolites within the gut environment is fundamental to determine the applicability of PAC against enteric pathogens. Some mechanistic studies have shown that PAC have direct modulatory effects on immune cell signalling, isolated pathogens, and gut mucosal barrier integrity. Boosting the recruitment of immune cells and suppressing the amount of pro-inflammatory cytokines are modulating factors regulated by PAC, and can either be beneficial or detrimental in the course of re-establishing gut homeostasis. Herein, we review how PAC may alter distinct immune responses towards enteric bacterial, viral and parasitic infections, and how the modulation of gut microbiota may act as a mediating factor. Furthermore, we discuss how future studies could help unravel the role of PAC in preventing and/or alleviating intestinal inflammation and dysbiosis caused by enteric disease.

## Introduction

The mammalian gut environment is an intricate ecosystem where multiple components such as epithelial cells, immune cells and the gut microbiota (GM) interact reciprocally with each other and regulate homeostasis (1). The gut plays an important role in digesting and absorbing nutrients, preventing loss of water and electrolytes, tissue injury repair, and communicating with the environment. Moreover, it continually encounters a large amount of exogenous agents derived from the diet and environment, as well as infectious microbes (2). Pathogenic agents such as bacteria, parasites and viruses are a major health and socio-economic burden in both humans and livestock (3, 4). A major task of the gastrointestinal ecosystem is to maintain homeostasis in the presence of harmless dietary components and commensal microbiota, whilst eliciting an adequate protective inflammatory response in response to pathogens (5). Understanding this delicate balance may be the key to developing novel interventions for control of infectious diseases. This is of critical importance, as new approaches to prevent enteric infections in human and veterinary medicine are urgently required in the face of rising antimicrobial drug resistance (6).

The activities of the gut immune system may be highly responsive to diet, and it is increasingly appreciated that various dietary components can modulate the gut immune environment by directly or indirectly interacting with gut immune cells (7). Consistent with this, various natural products such as fibres, polyphenols, and other plant secondary metabolites have been investigated for their ability to regulate intestinal inflammation and immune function (8, 9). In particular, much recent attention has focused on the proanthocyanidins (PAC), a diverse class of oligomeric and polymeric polyphenols found in a wide range of foods including fruits, nuts and berries (10). PAC have been identified as the fraction most associated with the beneficial effects of fruits and berry extracts on diabetes and obesity (11). Whilst these positive effects of PAC are well-known in context of metabolic diseases, less is known about the role in PAC in regulating gut immunity to intestinal pathogen infection. Moreover, although our understanding of the health benefits of PAC has increased, precisely how PAC modulate the immune system is still mostly unknown. In this review, we examine the immunomodulatory effects of dietary PAC, as well as emerging evidence that PAC may modulate immune function during enteric infection in both humans and animals. We discuss current knowledge on the mechanisms underpinning the effects of PAC on inflammation and immunity, and highlight key studies showing that PAC may play a role in promoting resistance to pathogen infection and/or supressing immunopathology. Finally, we suggest some pertinent areas for future investigation.

## Gut Pathogens and the Mucosal Immune System

Worldwide, enteric infections are a major cause of mortality and morbidity in humans and production animals. In low and middle income countries, infections of the gastrointestinal tract including bacterial infections (e.g. *Escherichia coli)*, parasitic infections (e.g. hookworm) or viral infections (e.g. rotavirus) cause widespread disease and stunt socio-economic development (12). These infections are less common in high income countries but infections with pathogens such as *Clostridioides difficile* still pose a considerable public health burden (13). Notably, lifestyle factors such as poor diet (high sugar/high fat) and lack of exercise may not only lead to metabolic diseases, such as type 2-diabetes, but may also predispose to opportunistic infections due to dysfunctional mucosal barrier function (14). Enteric infections are also a major constraint in all livestock production systems, causing billions of dollars in lost production annually and compromising animal welfare and food security (15). Importantly, rising resistance to antimicrobial drug treatments in both humans and animals has necessitated the need for novel solutions for promoting healthy gut function and disease resistance (16).

The gut immune system plays a key role in regulating infection, not only by driving protective immune responses that remove pathogens but also co-ordinating wound healing, tissue repair and resolution of inflammatory cytokines to ensure the avoidance of harmful immunopathology. Under homeostatic conditions, the gut mucosal immune system is inherently programmed to be immunologically tolerant towards food antigens and commensal organisms. Loss of this immunological tolerance can result in dysregulated immune responses, which contributes to the development of inflammatory diseases (1, 17).

Anatomically, the gut immune system can be divided into three different components: the intestinal epithelial barrier (IEB), the lamina propria (LP) and the gut-associated lymphoid tissue (GALT), which is further comprised of Peyer’s patches (PP), isolated lymphoid follicles, and mesenteric lymph nodes (MLNs) (18). The IEB is comprised of diverse and specialized sets of integrated epithelial cells. At the mucosal surface, enterocytes are tightly joined by well-regulated intercellular junctional complexes, which prevents access by pro-inflammatory agents to the underlying tissue. The epithelium also comprises intra-epithelial lymphocytes (IEL), goblet cells, Paneth cells and enteroendocrine cells among others, which are assembled into a single layer covered by a mucous layer (19). Goblet and Paneth cells secrete mucus, and anti-microbial peptides, respectively, which contribute to the exclusion of allochthonous microbes from the LP tissue. Furthermore, IEL such as γδ T-cells help to maintain barrier integrity by promoting tolerance to commensal microbes, while responding to the presence of pathogens (20).

Within the LP, innate immune cells are uniquely situated to counter infectious microorganisms. These include rapid-responding innate lymphoid cells (ILCs), and professional antigen presenting cells (APCs) such as dendritic cells (DCs) and macrophages. The adaptive arm of gut immune system is constituted by B, T and antibody-secreting plasma cells typically arranged within both the LP and the GALT (18). Macrophages and DCs possess several pattern recognition receptors, including toll-like receptors (TLRs), and C-type lectin receptors, that are capable of recognizing pathogen-associated molecular patterns (21). Both the innate and adaptive arms of the gut immune system work in a coordinated manner to maintain immunologic homeostasis and induce protective inflammatory responses (22, 23). Exogenous antigens are sensed by APCs that possess the specialized machinery to sample and process incoming antigens. For the induction of the gut immune response antigen-bearing DCs migrate to the nearby GALT tissue like PP or MLN where DCs communicate with the naïve T-cells and induce their antigen-specific differentiation into effector T cells which can be either CD4^+^ [T-helper (Th) 1, Th2, Th17, regulatory T cells (Treg)] and/or CD8^+^ (cytotoxic) T cells (24). ILCs are also involved in initiating immune responses (23). After induction, the effector immune cells either remain in the GALT tissue or migrate to the suitable mucosal sites where they induce protective inflammatory responses resulting in the elimination of the antigen and the re-establishment of homeostasis.

Depending upon the nature of the pathogen challenge, gut immune responses can be classified into subtypes; type 1 immune responses (comprising mainly ILC1 and Th1 cells) that provide protection against viral and intracellular bacteria, type 2 immune responses (ILC2 and Th2) that help in expelling parasitic helminths, and type 3 immune responses (ILC3 and Th17-Th22) that are required to eliminate fungi and extracellular bacteria (24). Furthermore, an additional type 4 immune response has been suggested to operate at the gut mucosal surfaces that principally block the entry of microorganisms even before they come in contact with the mucosal surfaces. This response is mediated by secretory IgA, which is in turn dependent on the production of TGFβ secretion from Treg cells (25, 26). Regulation of these different pathogen-specific immune programs is crucial. Dysregulation during either chronic infection or autoimmune disease can lead to Th1 and Th17-driven chronic inflammatory diseases like Crohn’s disease (27), whereas impaired Th2 immune responses contributes to the progression of food allergies (28) and ulcerative colitis (29). Thus, the effects of immune-modulating dietary interventions (e.g. prebiotics) need to be evaluated not only on how they may boost immune responses to infection, but also how they impact on the resolution of inflammation and regeneration of tissue damage. Ultimately, nutritional manipulation of the gut immune system should aim to promote innate and adaptive responses that allow infected hosts to resist and/or tolerate infections, with a minimum of immunopathology.

## Proanthocyanidins

Proanthocyanidins are a member of the polyphenol class of plant secondary metabolites. Polyphenols represent an important group of naturally occurring anti-oxidants and chemo-preventive compounds, and are found in plants and in food of plant origin, such as fruits, vegetables, cereals, and cocoa. The average intake of polyphenols by European adults has been estimated to ~1 g/day, which is higher than the intake of any other classes of phytochemicals (30, 31). The main challenge that delayed the attention to polyphenols compared to other anti-oxidants, such as carotenoids and selenium, was due to the considerable diversity and complexity of their chemical characteristics (30). Polyphenols have especially been studied to investigate their potential in cancer research due to their anti-oxidative effects, but their mechanisms of action go beyond the modulation of oxidative stress, and are not yet fully understood (30, 32). Epidemiological, *in vitro* and animal studies have shown that polyphenols may have preventive and protective properties towards numerous conditions, such as cardiovascular, degenerative, metabolic, and inflammatory diseases (33, 34).

**Figure 1:**
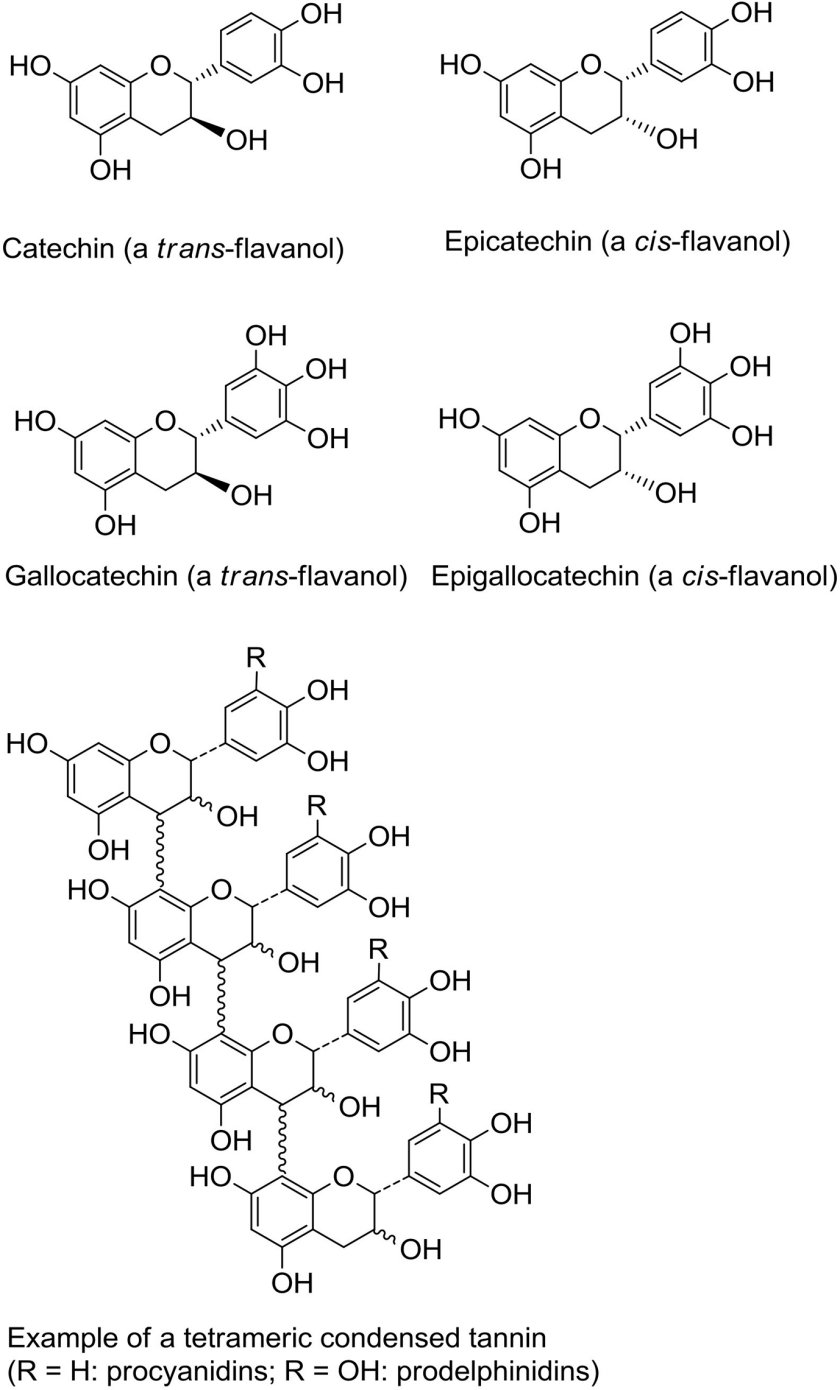
Structures of flavanol monomeric subunits and an example of a tetrameric proanthocyanidin (condensed tannin) oligomer.

One of the most widespread polyphenol classes are tannins: water-soluble secondary metabolites found in many different plants. They can be classified into two main groups; the hydrolysable tannins (gallotannins, ellagitannins, and complex tannins) and the non-hydrolysable PAC, or condensed tannins (35). Proanthocyanidins consist of flavan-3-ol monomers, either catechin or epicatechin or their *trans* isomers, arranged into large oligomers or polymers with degrees of polymerization (DP) ranging from 2 to more than 40 (**Figure 1**) (36, 37). They are among the most common dietary polyphenols and are responsible for the bitter taste of chocolate and the astringent sensation in fruits such as grapes, pears and apples. The sensation of astringency is caused by the ability of PAC to bind and precipitate salivary proteins containing high-proline contents (38). The average intake of PAC has been estimated to 95 mg/day in American adults with variations depending on age, sex, wine consumption and ethnicity (39). Of note, due to large variation in PAC DP, no food source can provide high amounts of PAC molecules with one specific chemical conformation (40). Thus, the studied effects of PAC towards a vast variety of diseases often relies on a symbiotic or additive effect of PAC molecules with differing DP.

### Gut Immune Regulation by Dietary Proanthocyanidins

Polyphenols (including PAC) display π-electron-rich aromatic nuclei and labile phenolic –OH groups, which confer them a reducing (electron and hydrogen-donating) character. Thus, their antioxidant effects are due to their ability to rapidly reduce reactive oxygen or nitrogen species (ROS/RNS), which are produced in great amounts during the inflammatory phase of chronic diseases (41). In addition, the PAC molecule procyanidin B1 (a dimer of epicatechin) has also shown protective effects towards oxidative stress by inducing the activity of the glutathione S-transferase P1 enzyme (GSTP1) and the nuclear translocation of the transcription factor NF-erythroid 2-related factor (Nrf2), which may in fact be a more important antioxidant mechanism than direct scavenging of ROS (42). Due to their well-known antioxidant effects, and their high concentrations in many health-promoting dietary components such as grapes and berries, PAC have been increasingly studied for their effects on gut health. Numerous studies in mouse and piglet models of intestinal inflammation have shown that dietary PAC can have beneficial effects, including promoting gut barrier integrity and mucosal morphology, and increasing goblet cell density and villi lengths (43, 44). However, the mechanisms by which PAC induce these benefits are largely unknown.

PAC molecules generally remain stable in the stomach (45, 46) which may be due to a protective layer formed by the protein-binding oligomers coating the gastric mucosa or by modulating hydrochloric acid secretion (47). Thus, PAC with a DP of ≥3 remain structurally intact during intestinal transit, and are able to pass to the large intestine where they are metabolized to varying degrees by the GM. This results in the production of numerous bioavailable metabolites such as flavan-3-ol conjugates (e.g. O-methyl-epicatechin-glucuronide) and phenolic acids (e.g. caffeic or coumaric acids). These can be absorbed and result in systemic health benefits, such as positive regulation of cardiovascular disease by binding to low density lipoproteins (48–51).

**Figure 2:**
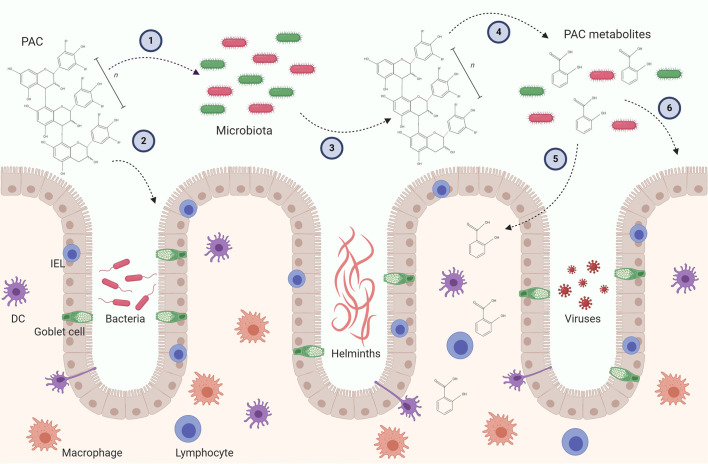
Possible mechanism leading to immunomodulatory activity of proanthocyanidins (PAC) in the gut. PAC molecules enter the gut lumen and may exert direct effect on the gut microbiota by selecting for distinct bacteria (1) and the gut epithelial layer by upregulating i.e. tight junctions (2). In turn, the gut microbiota may alter the chemical structure of the parent PAC molecule and produce metabolites (4). PAC metabolites can be absorbed through the epithelial layer and influence residing immune cells (5). The metabolites may also alter gut microbiota as well as the gut epithelial barrier (6). Furthermore, both parent compounds and PAC metabolites may have direct or indirect effects on gut pathogens, such as bacteria, helminths and viruses. These putative immunomodulatory mechanisms may be caused by isolated PAC molecules and/or PAC-rich extracts. Conclusive experimental evidence *in vivo* is thus far lacking. DC, Dendritic cell; IEL, Intraepithelial lymphocyte.

A number of recent studies have begun to shed light on how microbial metabolism of PAC may influence gut health. Some caution needs to be applied in ascribing the observed effects solely to PAC, as feeding studies are often conducted with extracts or dietary supplements which contain other polyphenols or fibres which may also impact the GM. Of interest, it has been shown that transfer of GM from mice fed a PAC-rich *Camu camu* extract mice to germ-free mice can protect against metabolic disease during diet-induced obesity (52). Thus, a prebiotic effect of PAC has been speculated to be primarily responsible for their anti-inflammatory and immunomodulatory activity (8, 53). Consistent with this, changes in the GM following administration of pure grape-seed PAC can precede alterations in intestinal immune gene expression (54). However, it is also plausible that PAC interact directly with cells at the mucosal barrier during their intestinal transit, and this may also result in significant immunomodulatory activity (55). Consequently, the effects of PAC on the immune system likely comprise both prebiotic effects and direct modulation of immune cell function (**Figure 2**). We will discuss both these potential mechanisms below.

### Regulation of Immune Function by Proanthocyanidins as a Result of Prebiotic Effects

The GM residing in the human intestines is composed of up to 100 trillion microbes (56). Most of the intestinal bacteria belong to different genera of gram-positive Firmicutes, and to some of the many different gram-negative Bacteroidetes, such as *Bacteroides, Prevotella, Parabacteroides*, and *Alistipes*. Other phyla, including *Proteobacteria, Actinobacteria, Fusobacteria, Verrucomicrobia* are also core members of the human GM (57). The number and diversity of bacteria have been shown to vary in the different sections of the gastrointestinal tract. Thus, a low number and few species populate the stomach and the upper part of the small intestine, whereas the number of bacteria progresses from the jejunum to the colon (58). Recently, the concept of “healthy gut” has become very popular given that intestinal dysfunction has been associated with several diseases, both locally as well as systemically (1). Furthermore, perturbed gut immune homeostasis also weakens the gut barrier integrity, increasing susceptibility to opportunistic pathogen infection and allowing gut bacterial translocation to the basal side of the mucosa, resulting in systemic inflammation (5, 59).

A number of studies have shown that dietary PAC can alter the composition of the GM. Piglets given grape-seed derived PAC had improved gut microbial diversity, and increased abundance of OTUs belonging to *Firmicutes*, *Bacteroidetes* spp., and *Clostridiaceae*, and decreasing the abundance of *Lactobacillaceae* (44). Moreover, a consistent feature of PAC-rich diets is a significant increase in the abundance of *Akkermansia muciniphila*, which has been observed in mice, pigs and humans (60–62). *Akkermansia* has become a biomarker for gut health due to its association with mucosal barrier integrity and mucin production. It can also produce metabolites that suppress inflammatory responses directly in intestinal epithelial cells, suggesting that its growth in response to certain dietary components may be responsible for their putative health benefits (63). Various other metabolites with known anti-inflammatory activity, such as short-chain fatty acids (particularly propionate), have also been observed to be increased in the digesta of animals fed PAC-rich diets (61, 64).

How PAC causes these changes in the GM is an open question and an active area of research. Several studies have addressed the metabolism by which PAC molecules are degraded into aromatic acids by the intestinal flora, notably in the large intestines (33). It is clear that, at least *in vitro*, the bacterial metabolism of PAC is dependent on the DP, with polymers being less susceptible to degradation compared to catechin monomers (65). Stimulated digestion of PAC by the GM in *in vitro* systems has demonstrated an active de-polymerization of PAC followed by the appearance of small phenolic metabolites, some corresponding to those observed in the systemic circulation of animals fed PAC *in vivo* (66, 67). Thus, it is clear that PAC can act as a direct prebiotic substrate, similar to dietary fibre. Furthermore, direct anti-bacterial effects of PAC have long been studied, and it is known that PAC induce growth inhibition of some bacteria either by inhibition of enzymes, deprivation of growth substrates, or direct action on the bacterial cell membrane (68). Therefore, selective inhibition of some bacterial taxa, allowing the increased propagation of beneficial bacteria, may also be a mechanism whereby PAC change the composition of the GM. Indeed, selection for *Bacteroides* and *Enterobacteriaceae* is seen in rats fed a PAC-rich diet, and these bacteria were referred to as PAC-resistant bacteria, and their abundance increased in a dose-dependent manner (69). However, it is also clear from *in vitro* studies that direct interactions between PAC and host intestinal cells can stimulate the production of mucins and other mucosal proteins, that may also act as selective nutrient source for bacteria such as *Akkermansia* (70). Consequently, growth of these bacteria may indirectly derive from PAC-mediated effects on host cells rather than a direct interaction between PAC and the bacteria residing within the GM. Whatever the mechanism, it is clear that the consumption of PAC changes the GM and produces soluble metabolites with recognized anti-inflammatory or immunomodulatory activity. This prebiotic capacity of PAC may thus be a major mechanism of their observed health benefits in different models of disease.

### Direct Effects of Proanthocyanidins on Immune Cells

*In vitro*, PAC possess well-characterized anti-inflammatory activity in both intestinal epithelial cells, macrophages and DCs (71, 72). Exposure of macrophages or DCs to pro-inflammatory stimuli such as TLR ligands (e.g. lipopolysaccharide—LPS) in the presence of PAC results in lower production of inflammatory cytokines, ROS, and NFκB translocation (55, 73). Moreover, in epithelial cells, mitochondrial dysfunction induced by inflammation and oxidative stress can be effectively alleviated by PAC (74). Mechanistically, the mode-of-action of PAC has not been fully elucidated. However, PAC do not appear to block the interaction of LPS with TLR4 in DCs, but instead modulate downstream signalling pathways connected to lysosome activity and second-messenger activity (73, 75). Importantly, these effects are consistent with immunological changes observed *in vivo* in models of inflammation, suggesting that at least some anti-inflammatory effects of PAC derive from direct modulation of mucosal immune cells (76–78).

In addition to regulating cellular responses to pro-inflammatory stimuli, PAC are also capable of activating innate immune cells involved in host defence and barrier function. Perhaps the most well described example of this is the *in vitro* response of γδ T-cells to PAC stimulation, whereby these cells undergo proliferation, up-regulate IL-2Rα and display increased stability of transcripts encoding for cellular activation (79, 80). This response appears to be unique to γδ (and not αβ) T-cells, is conserved across multiple species, and has also been observed *in vivo* in humans consuming PAC-rich cranberry juice (81–83). γδ T-cells are found in the intestinal barrier where they respond to conserved pathogen or dietary antigens to fortify mucosal defences and provide signals for the activation of other immune cells such as neutrophils (84). Thus, these cells appear to have evolved a conserved response against PAC, suggesting that intake of these dietary compounds is associated with activation of innate defence mechanisms leading to protection from infection. Consistent with this, stimulation of intestinal organoids with PAC leads to significant up-regulation of antimicrobial defences (e.g. goblet cell and mucin production), again indicating that the mammalian gut has evolved to sense PAC as a signal for strengthening of innate immune defences to prevent infection and/or reduce harmful inflammation (70).

### Effects of Proanthocyanidins on Resistance to Enteric Pathogen Infection

A major research effort is currently underway to identify nutritional interventions that can improve resistance to infection in both human and veterinary medicine. Nutritional manipulation of the immune system may enhance protective response to infection but also reduce inflammation and restore homeostasis and tolerogenic immune responses. Much of this research has been directed towards probiotics, and the role that they may play in stimulating the immune system and promoting balanced immune function without overt inflammation (85). The ability of PAC to increase resistance or modulate inflammatory responses to infection is not yet clear. Below, we consider some relevant studies where the effects of PAC on different enteric infections have been studied.

#### Bacterial Infections

The colonization and invasion of pathogenic bacteria leads to the disruption of gut homeostasis, inflammation and often symptomatic disease. Whilst dietary PAC can protect against pro-inflammatory responses induced by either diet-induced GM dysbiosis or acute challenge with purified endotoxin (86), their role in combating infection with bacterial pathogens is less well described. As mentioned above, PAC have documented anti-bacterial effects which include preventing the entry of *E. coli* into epithelial cells, likely by Fimbriae neutralization or agglutination, and also deactivation of toxins which can cause secretory diarrhoea (70, 87). This may explain the apparent clinical benefits of feeding PAC-rich extracts to piglets infected with enterotoxigenic *E. coli* (88). Moreover, some of the immunomodulatory effects that are apparent during metabolic disease models, (e.g. strengthening of the mucosal barrier by goblet cell differentiation and modulating inflammatory cytokine production), also appear to be relevant in different bacterial infection models. Peptic ulcers are one of the leading gastro-intestinal diseases and can often be attributed by the gram-negative bacteria *Helicobacter pylori*, which affects the duodenum and stomach by inducing inflammation, and increasing the risk of adenocarcinoma (89). A number of studies have investigated how PAC may alleviate infections with *H. pylori.* In a rat model of gastric ulcers, the beneficial modes of actions of PAC were attributed to elevated mucus secretion, increased recruitment of neutrophils and mast cells, as well as a thicker regenerative gastric mucosa (90). In pigs, provision of grapeseed PAC reduced infection with *Campylobacter jejuni*, with the effects ascribed to enhanced mucosal barrier function as a result of reduced ROS, and consequently less disruption of epithelial tight junctions (91). In addition, the anaerobic and gram-positive bacteria *Clostridium perfringens* is the causative agent of necrotic enteritis in poultry. It primarily affects the jejunum and ileum, and causes important economic losses in the poultry industry (92). A study using concomitant infection of both coccidia and *C. perfringens*, demonstrated a significant decrease in the amount of intestinal lesions and mortality, when feeding PAC as a boosting agent to increase the efficacy of a vaccine (93), with the authors hypothesizing that PAC may be capable of simulating both cytotoxic and T-helper cells during infection.

Whilst these studies have demonstrated that dietary PAC may help to protect against pathogenic bacterial infections, Forgie *et al. (* 94 *)* recently reported that mice fed a PAC-rich diet were more susceptible to infection with *Citrobacter rodentium* (a model for *E. coli* infection in humans). These authors speculated that a reduction in GM diversity stemming from PAC consumption deprived the mucosal barrier of protection from commensal bacteria that could prevent *C. rodentium* attachment. In addition, it could be postulated that PAC may inhibit the production of inflammatory cytokines, such as IL-6, which are important for defense against *C. rodentium* (73, 95). Thus, the immunomodulatory effects of PAC could, in some contexts, render hosts more susceptible to infection if a strong inflammatory response is necessary to clear the pathogen. Thus, careful appraisal of the effects of PAC in different host-pathogen systems is required to determine their potential benefits. Interestingly, treatment of mice with extracts from pomegranate that contain hydrolysable tannins (as opposed to PAC, or condensed tannins) reduced *C. rodentium* infection and also appeared to alleviate some of the dysbiotic effects induced by the infection (96). Thus, exploration of structure-function relationships of different polyphenols and how these relate to regulation of responses to bacterial infections are clearly required. Moreover, studies to determine whether there are differences in the ability of PAC to regulate chronic, low-grade infections, or acute infections accompanied by significant epithelial inflammation are highly warranted.

#### Parasitic Infections

Recent estimates are that more than a billion people are infected by intestinal worms (helminths), making parasite infections one of the most common infections worldwide, and causing substantial morbidity (97). Parasitic infections also cause significant economic losses in farming industries. Helminths are also ubiquitous in livestock where they cause reduced performance and as well as clinical disease (98). In addition, protozoan parasites such as *Giardia* are extremely common in tropical regions, and related parasites such as *Eimeria* are the cause of coccidiosis which is a major cause of clinical diarrhoea in animals such as chickens and calves (99). Unlike bacteria and viruses, immunity to helminth infection is critically dependent on Th2-driven immune responses. IgE production, eosinophilia and production of type-2 cytokines such as IL-13 and IL-33 are all hallmarks of helminth infection (100). In natural infections, mixed Th1/Th2 responses are induced. Modulation of this balance can either induce Th1-driven immunopathology with tissue necrosis, pro-inflammatory responses and chronic infection, or Th2-related protective immunity involving remodeling of the intestinal barrier and mucus secretion that results in parasite expulsion (101).

It has been repeatedly shown that high levels of PAC in the diet may help animals cope with helminth infection. Interestingly, animals have a natural preference for specific plants when infected with parasites, and self-medicate by ingesting secondary plant metabolites, including PAC (102). Worms recovered from sheep or cattle consuming forage pastures rich in PAC (e.g. from the Fabaceae family) are smaller and less fecund than worms from control-fed animals, indicating a beneficial effect of PAC on response to infection (103), and *in vitro* experiments have shown that PAC can directly bind to helminths and reduce their survival (104, 105). More recently, a number of studies have shown that PAC can substantially alter the immune response during helminth infection, suggesting that host resistance to infection can be enhanced. *In vitro*, DCs exposed to PAC have an enhanced ability to drive helminth-induced Th2 responses in naïve T-cells, suggesting that parasite-specific immune function can be enhanced during infection (73). Consistent with this, helminth-infected animals consuming PAC have higher numbers of Th2-associated mucosal eosinophils and mast cells, as well as parasite-specific antibodies and γδ T-cells (64, 106–110). Heightened Th2 polarization may result from the selective down-regulation of pro-inflammatory Th1 responses by PAC (73). Alternatively, direct stimulation of gut epithelial cells, either by parent PAC molecules or GM-derived metabolites, may enhance innate defenses that favor anti-helminth immunity (e.g. goblet cell responses). Beneficial effects of PAC are not confined only to helminth parasites. Poultry infected with *E. tenella* and fed with PAC have significantly decreased mortality rates and increased weight gain, which was linked to amelioration of oxidative stress caused by infection (111).

Collectively, these studies suggest that PAC may offer multifaceted benefits to parasitized hosts. These may include a reduction in parasite fitness, as well as augmentation of immune responses that may suppress harmful inflammation, oxidative stress and pathology, whilst promoting immune responses that fortify and repair the mucosal barrier, thus protecting from secondary bacterial infections that are also a feature of worm infections (112). Further research is needed to understand these effects, and helminth infection models may offer a valuable model for assessing the ability of PAC to modulate naturally induced type-2 mucosal immune responses.

#### Viral Infections

Enteric viruses are mainly transmitted *via* the fecal–oral route, either by person-to-person contact or by the ingestion of contaminated food or water (113). The most important viruses causing gastroenteritis in humans include norovirus and hepatitis A (114). In addition, sapoviruses, rotaviruses, coronavirus, astroviruses, and hepatitis E virus (HEV), are also known causes for enteric diseases (113).

Research into the potential role of PAC on combating enteric viral infections is in its infancy. Several studies have investigated the ability of PAC to neutralize viruses *in vitro.* PAC derived from persimmon were shown to inactivate 12 pathogenic viruses, including rotavirus (115). Similar effects with other PAC preparations have been established in other *in vitro* studies, which demonstrated anti-viral effects of PAC against the norovirus surrogates Bacteriophage MS2, murine norovirus (MNV-1), and Feline Calicivirus (FCV-19), as well as Hepatitis A and coxsackievirus (114, 116).

The ability of PAC to modulate immune responses to viral infection have also been investigated *in vitro.* Exposure of human PBMC infected with dengue virus to oligomeric PAC resulted in enhanced type-1 interferon responses, and lower viral titers, suggesting that PAC can directly impact on the intracellular response to viral infection (117). Consistent with this, consumption of apple-derived PAC by mice also resulted in augmented type-1 interferon activity (118). Some limited clinical evidence exists in support of this from a study where prolonged consumption of PAC-rich cranberry juice reduced influenza symptoms (83). These results imply that PAC have the ability to activate innate immune effector mechanisms that target intracellular viruses, similar to the triggering of defense molecules such as mucins in the gut mucosa, suggesting a highly conserved response whereby sensing of PAC leads to a rapid response designed to protect against pathogens. Importantly, PAC also appear to down-regulate excessive inflammatory cytokine production in response to viral infection which, in many cases, is responsible for the pathology and clinical symptoms (119). However, further work is clearly needed with detailed *in vivo* studies to explore if dietary PAC can effectively modulate immunity and inflammation during enteric viral infection.

## Concluding Remarks

A vast number of studies have now documented that, *in vitro*, PAC possess anti-oxidant, anti-bacterial, anti-parasitic, and anti-viral effects. Moreover, the ability of dietary PAC to modulate the GM and protect against obesity and metabolic syndrome is becoming well-established. Whilst there is clear evidence that dietary PAC affect immune function, our understanding of the implications of this is only just starting to be formed. The balance between effective immune responses and harmful immunopathology is a fine one. Dietary supplements that induce immunosuppressive effects may be beneficial for autoimmune disease, but may in turn be detrimental against infectious diseases by potentially giving rise to increased susceptibility of infection. Understanding the mechanisms leading to the anti-inflammatory effects of PAC may therefore lead to better decision-making on how/when to use PAC during aberrant immune responses towards pathogens, while not supressing the vital immune components required for overcoming infections. It has also become clear that development of infection is highly dependent on complex host-pathogen interactions, and future studies should consider the implications of PAC during the early and late stages of acute or chronic enteric disease. This would broaden the possible applications of PAC throughout the course of disease, as well as elicit further investigations into the modes of action of PAC as the gut homeostasis alters during disease progression. The possible benefits of PAC may also be explored prior to infection, to unravel its potential at priming the gut environment towards enhanced immunity, or post-infection where PAC may improve healing of the gut mucosal barrier.

Further understanding the mechanisms of how PAC modulate the activity of immune cells will be crucial to further optimize their use as health-promoting dietary additives. Key questions to be addressed include determining the specific pathways that are modulated in immune cells such as macrophages after exposure to PAC—does their anti-inflammatory activity stem purely from antioxidant effects or do PAC specifically alter key intracellular signalling pathways? How much of the observed immunomodulatory activity of PAC is due to direct effects of parent PAC compounds on mucosal immune cells, compared to the activity of GM-derived metabolites that are formed during breakdown of PAC in the colon? Which specific metabolites can alter the activity of immune cells and gut barrier function? Can PAC directly kill pathogens in the gut, or are beneficial effects purely related to effects on host barrier function and immunity? A detailed understanding of how PAC influence immune responses towards different pathogens that induce type-1 responses (intracellular bacteria), type-2 response (helminths), or type-3 responses (extracellular bacteria) will also be important if PAC-rich dietary components are to be strategically used to combat enteric infections in human or veterinary medicine. Indeed, PAC may prove to be a useful model for answering basic questions about the regulation of mucosal immunity by dietary compounds. However, the lack of standardized methods in the purification techniques of PAC limits the comparison of data between studies. Also, most *in-vivo* studies make use of PAC-rich extracts, which may give rise to misleading conclusions regarding the isolated effect of PAC. Future studies should therefore aim at finding a general consensus as to how to isolate chemically identical PAC molecules. This is crucial, as numerous studies have demonstrated that there are substantial differences in bioactivity of PAC subtypes (based on DP or monomeric subunits) (73, 74). Mechanistic studies could consequently attribute a specific mode of action to distinct PAC molecules. This would aid minimizing the considerable variability observed between research groups working with isolated PAC molecules of various purity compared to plant extracts rich in PAC among other phenolic compounds. Increasingly rigorous methods will need to be applied, including the use of germ-free animals and other experimental approaches to disentangle the role of the GM in the immunomodulatory activity.

In conclusion, PAC seem to be a promising group of natural dietary compounds that can regulate inflammation. Emerging evidence exists that mucosal immunity to pathogen infection can be enhanced by PAC. Specific mechanisms identified include the priming of innate defenses in mucosal epithelial cells and leukocytes as well as promoting antibody and balanced lymphocyte responses through regulation of inflammatory responses during infection. A multidisciplinary approach involving phytochemistry, microbiology and immunology will be necessary to understand the interactions between PAC and the immune system, and lead to the full exploitation of defined PAC molecules as health-promoting dietary agents.

## Author Contributions

AA-C, PA, and AW wrote the paper. AW revised the paper. All authors contributed to the article and approved the submitted version.

## Funding

The authors were supported by the Independent Research Fund Denmark (Grant # 7026-00094B). The funding body had no role in the preparation of the manuscript or decision to publish. 

## Conflict of Interest

The authors declare that the research was conducted in the absence of any commercial or financial relationships that could be construed as a potential conflict of interest.
